# Quantitative genetic analysis of the bTB diagnostic single intradermal comparative cervical test (SICCT)

**DOI:** 10.1186/s12711-016-0264-3

**Published:** 2016-11-24

**Authors:** Smaragda Tsairidou, Susan Brotherstone, Mike Coffey, Stephen C. Bishop, John A. Woolliams

**Affiliations:** 1 The Roslin Institute and R(D)SVS, University of Edinburgh, Easter Bush Campus, Midlothian, EH25 9RG Edinburgh, UK; 2 Institute of Evolutionary Biology, University of Edinburgh, King’s Buildings, West Mains Road, EH9 3JT Edinburgh, UK; 3 Animal and Veterinary Sciences, SRUC, Roslin Institute Building, Easter Bush Campus, Midlothian, EH25 9RG Edinburgh, UK

## Abstract

**Background:**

Bovine tuberculosis (bTB) is a disease of significant economic importance and is a persistent animal health problem with implications for public health worldwide. Control of bTB in the UK has relied on diagnosis through the single intradermal comparative cervical test (SICCT). However, limitations in the sensitivity of this test hinder successful eradication and the control of bTB remains a major challenge. Genetic selection for cattle that are more resistant to bTB infection can assist in bTB control. The aim of this study was to conduct a quantitative genetic analysis of SICCT measurements collected during bTB herd testing. Genetic selection for bTB resistance will be partially informed by SICCT-based diagnosis; therefore it is important to know whether, in addition to increasing bTB resistance, this might also alter genetically the epidemiological characteristics of SICCT.

**Results:**

Our main findings are that: (1) the SICCT test is robust at the genetic level, since its hierarchy and comparative nature provide substantial protection against random genetic changes that arise from genetic drift and from correlated responses among its components due to either natural or artificial selection; (2) the comparative nature of SICCT provides effective control for initial skin thickness and age-dependent differences; and (3) continuous variation in SICCT is only lowly heritable and has a weak correlation with SICCT positivity among healthy animals which was not significantly different from zero (*P* > 0.05). These emerging results demonstrate that genetic selection for bTB resistance is unlikely to change the probability of correctly identifying non-infected animals, i.e. the test’s specificity, while reducing the overall number of cases.

**Conclusions:**

This study cannot exclude all theoretical risks from selection on resistance to bTB infection but the role of SICCT in disease control is unlikely to be rapidly undermined, with any adverse correlated responses expected to be weak and slow, which allow them to be monitored and managed.

**Electronic supplementary material:**

The online version of this article (doi:10.1186/s12711-016-0264-3) contains supplementary material, which is available to authorized users.

## Background

BTB diagnosis in the UK continues to rely on the single intradermal comparative cervical test (SICCT) [1] and currently, ~93% of bTB cases have involved the use of SICCT at some point in the diagnostic process (Banos G., personal communication, November 18th, 2015). SICCT is a comparative test that measures skin thickness before and after simultaneous inoculation of the *M. bovis*-purified protein derivative (PPD) and *M. avium* sb sp*. avium*-PPD antigens, also referred to as tuberculins. By comparing the magnitude of the responses to the two tuberculins, and applying pre-determined thresholds to their difference, SICCT can differentiate most cases of true bTB infection from cross-reactions due to exposure to other mycobacteria in the environment [1]. SICCT is not a gold standard, and while it has very good specificity (*Sp*; *Sp* > 99%) [2, 3], its sensitivity is low (*Se*), with estimates ranging from 55 to 91% [1, 2, 4], with some of this variation depending on the protocol used for interpreting its values.

Previous genetic studies on confirmed bTB infection have demonstrated the feasibility of selection for bTB resistance in cattle, (a) by showing that there is heritable genetic variation for this trait [4–6], and (b) by providing initial estimates of the prediction accuracy for marker-based genomic selection [7]. Genetic selection for cattle that are more resistant to bTB can provide a complementary control tool to assist in reducing the within-herd bTB incidence, as well as the likelihood and severity of breakdowns [8]. However, SICCT-based screening plays a central role in bTB control in the UK, and will influence the identification of cases to be used for phenotypic best linear unbiased prediction (BLUP), or for training data in genomic selection. Therefore, critical questions arise as to what may be the impact of selection for bTB resistance on the properties of SICCT, i.e. whether in addition to increasing bTB resistance, correlated responses may alter the continuous distributions of SICCT responses in infected and uninfected cattle. This uncertainty hinders the use of genetic selection for resistance in the control of bTB.

Therefore, estimates of the extent of genetic variance and covariation in the continuous variation in SICCT response and its component responses to *M. bovis* and *M. avium* inoculations are a first step towards understanding the possible correlated responses to selection for bTB resistance. Key questions concern the possibility of changes in the *Se* and/or *Sp* of the SICCT test and the consequent risk of decreasing the effectiveness of an important tool for bTB control if these changes are unfavourable. Furthermore, if changes are predicted, how quickly might such changes emerge, and in which components of the SICCT test will they be expressed. For example, it has previously been hypothesised that the culling following regular SICCT screening will prompt changes in particular components of the SICCT [9].

The aim of this study was to conduct a quantitative genetic analysis of the continuous variation in the SICCT test using the extensive available field data collected during bTB herd testing. The genetic variances and covariances in response to the test inoculations are estimated for SICCT and its components, and the genetic basis of the hierarchical comparative structure of SICCT is explored. The study develops a quantitative genetic model to overcome some of the limitations of using field data, which arise from the uncertainty in identifying infected and healthy individuals unambiguously. This model is used to infer the potential magnitude of the genetic correlation between bTB resistance and the liability for positivity in healthy animals (i.e. the liability for being identified as a false positive), which is related to the individual *Sp*. Estimated breeding values (EBV) for bTB resistance in cattle are derived from the genetic evaluation of observed cases and survivors within a completed herd breakdown (a discrete epidemic contained within a herd), following the model of [5]. The continuous variation observed in the SICCT test studied here will have no direct role in this evaluation model although, as justified above, it is relevant to the problems of bTB control.

## Methods

### Origin of data

The data comprised 117,356 Holstein–Friesian female cattle, all with known sires, with 130,626 SICCT records originating from 646 herds undergoing bTB breakdowns. A breakdown is a period of strict control for an entire herd, starting when a case of bTB is identified in the herd and continuing until the herd is free of bTB as determined by the testing protocols. All animals had been tested over a period of 9 years (2002–2010), from herds undergoing their 1st, 2nd, 3rd or 4th bTB breakdown, with the majority of animals being tested during the 1st breakdown within a herd (*n* = 112,116). The age of the animals tested ranged from 43 to 6605 days, with a mean of 1397 days. Repeated measurements were available for 11,910 animals: 10,678 animals with two records, 1104 with three records, and 128 with four records.

### SICCT as a diagnostic tool

**Fig. 1:**
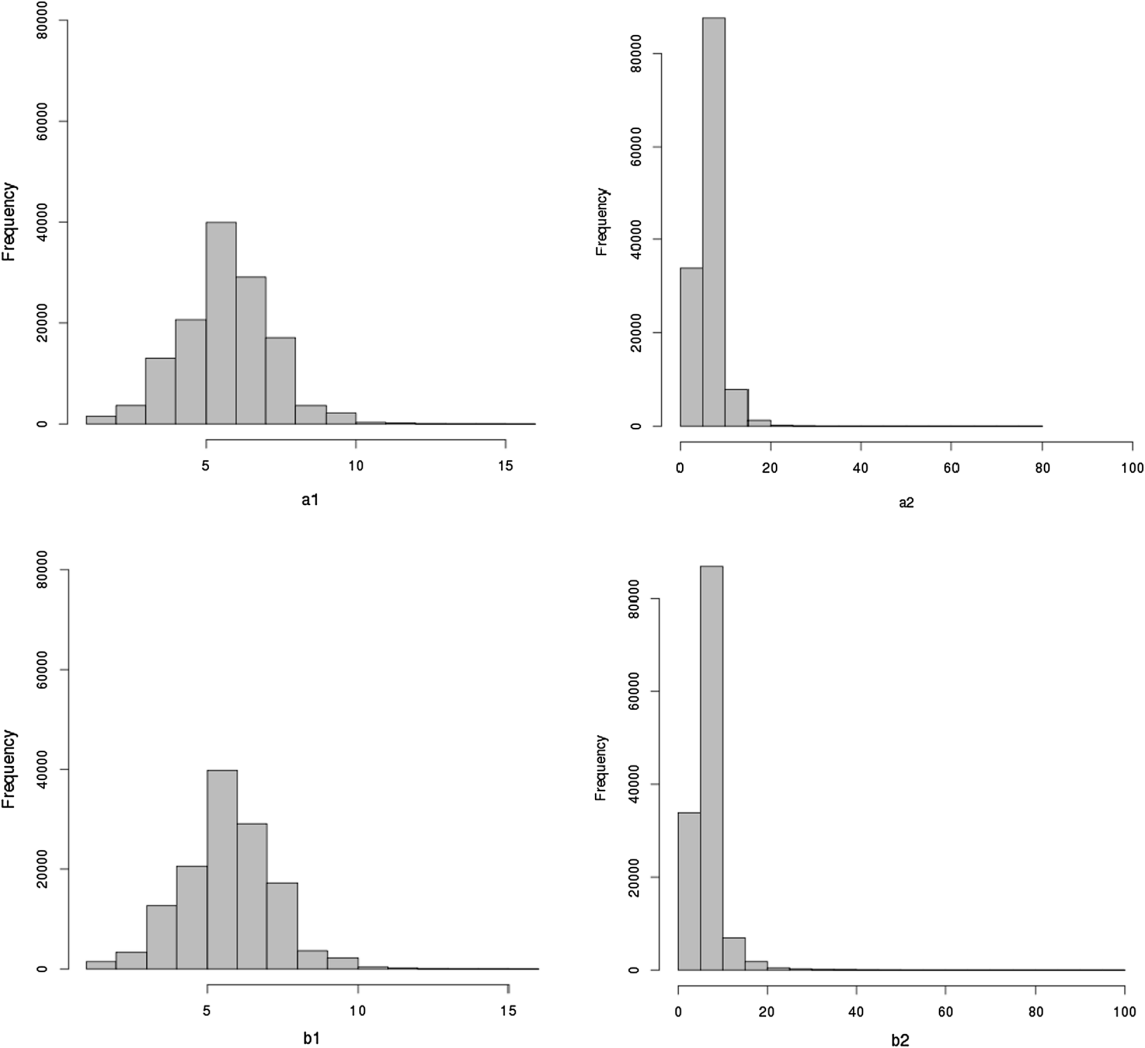
Histograms of SICCT component measurements. Frequency distributions for the individual skin thickness measurements, before (*a*_1_ and *b*_1_), and after (*a*_2_ and *b*_2_) inoculation of the tuberculins. *Note* the change in the x-axis scale for *a*_2_ and *b*_2_

SICCT involves four skin thickness measurements (mm): taken at the site of avian tuberculin injection before inoculation (*a*_1_) and 3 days post-inoculation (*a*_2_); at the site of bovine tuberculin injection before inoculation (*b*_1_) and 3 days post-inoculation (*b*_2_). A histogram of these measures is in Fig. 1. Three derived traits are calculated: (1) *da* = *a*_2_ − *a*_1_, which captures the responsiveness to *M. avium* inoculation; (2) *db* = *b*_2_ − *b*_1_ which captures the responsiveness to *M. bovis* inoculation; and (3) the test measurement for decision-making which is defined as *dc* = *db* − *da.* Individuals are categorised as non-reactors (NR), inconclusive reactors (IR), or positive reactors (R), following the standard or severe interpretations [10]. For the standard interpretation considered here, *dc* < 1 mm → NR, 1 ≤ *dc* ≤ 4 mm → IR, and *dc* > 4 mm → R. Individuals classified as NR and IR are treated as healthy, however, these may also include some misclassified bTB cases (i.e. false negatives) due to the imperfect *Se* of SICCT. This binary classification is decribed as SICCT positivity in this study, defined as 1 if R, or 0 if NR or IR under the standard interpretation. Those classified as R are considered to be cases and are culled, but in UK only a proportion of these cases undergo a confirmation process involving the culture of *M. bovis*, and some uncertainty remains over whether the remaining R are true or false positives. Since R are culled, the 11,910 repeated measurements are all NR or IR up to their final measurement.

### Quality control

Two animals with extreme *a*_2_ and *b*_2_ measurements of 77 and 99, respectively (considered to be wrong entries) were removed. Furthermore, the data indicated that 12 animals were retested although diagnosed as R, and the 25 records from these animals were removed. This left, 130,599 records for 117,342 animals that were retained for subsequent analyses. Assessment of the individual *a*_1_, *a*_2_, *b*_1_, and *b*_2_ measurements indicated that 11 herds with no standard R and less than 2 IR involving 418 records, but these were retained in the data. A detailed description of the quantitative data used in the analysis is in Table 1.

**Table 1:** Descriptive statistics for the components of SICCT after quality control

	Min	Median	Max	Mean	Var	SD
*a*_1_	1	6	16	6.17	2.33	1.53
*a*_2_	0	6	50	6.83	6.31	2.51
*b*_1_	1	6	16	6.19	2.33	1.53
*b*_2_	0	6	90	6.97	10.46	3.23
*da*	−7	0	43	0.66	3.54	1.88
*db*	−7	0	81	0.78	7.53	2.74
*dc*	−43	0	81	0.12	5.72	2.39

Descriptive statistics for the four skin thickness measurements, before (*a*_1_ and *b*_1_), and after (*a*_2_ and *b*_2_) inoculation of the tuberculins, and for the derived traits *da* = *a*_2_ − *a*_1_, *db* = *b*_2_ − *b*_1_, and the skin test *dc* = *db* − *da* following quality control

### Pedigree

**Fig. 2:**
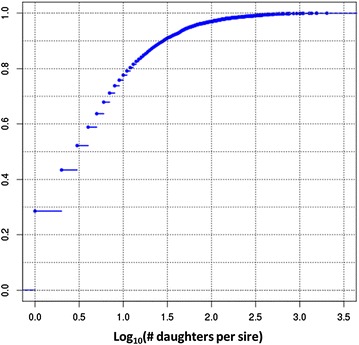
Daughters per sire distribution. Cumulative distribution of the number of daughters per sire for the total number of sires

The animals in the data were offspring of 7714 sires, of which 5510 had more than one daughter. The quartiles for the daughters per sire were *Q*_0_ = *Q*_1_ = 1, *Q*_2_ = 3, *Q*_3_ = 9, and *Q*_4_ = 2028, i.e. the median was equal to 3 (Fig. 2). Pedigree with both sire and dam known was available for 7376 of those sires. Pedigree completeness was assessed by the number of equivalent generations calculated using the ENDOG software [11] and was estimated at 4.27 in the pedigree provided.

### Preliminary analysis

The dataset for the preliminary analysis was constructed by retaining only the first record for each animal to avoid biases from the selective culling of R cases, and the further complexity of building adequate models for the behaviour of repeated records. This left 117,342 records in the analysis, from 728 breakdowns, containing 2704 standard R cases. Locally weighted regression (LOESS) on age was conducted on *da*, *db* and *dc* for the R cases using ‘R’ (R version 2.15.2) [12]. There was evidence of a pattern for animals younger than 3 years for *db* but not for *da* (see Additional file 1: Figure S1), and this influenced the design of the analysis across-ages.

### Detailed analyses

The detailed analysis was conducted on a dataset that was further reduced to contain only the first known test date within each of the 728 breakdowns and ignoring later tests. The justification for this is given in the “Results” section following the unrealistic outcomes of fitting model (1) as described below with an additional term to account for time trends in *dc*. This further reduction left 88,932 records in the analysis, containing 1261 standard R cases, with apparent prevalence *p′* = 1.4%. Additional file 2 explores the potential extent of false positives and false negatives in these data using literature values for *Se* and *Sp*.

#### Linear mixed models

Linear mixed models were fitted using ASReml [13]. Analyses comprised: (a) estimation of heritabilities for all SICCT measurements; (b) investigation of the genetic and environmental correlations and regressions between the components of SICCT; (c) estimation of the correlation between the *dc* measurement in healthy animals and SICCT positivity; and (d) exploration of the impact of age on the estimated heritabilities.

#### Univariate sire models

These were fitted in ASReml for the response variables *a*_1_, *b*_1_, *da*, *db*, and *dc* as follows:

1 $${\mathbf{y}} = {\mathbf{m1}} + {\mathbf{X\varvec{\upbeta} }} + {\mathbf{f}}\left( {\mathbf{t}} \right) + {\mathbf{Zu}} + {\mathbf{e}},$$

where ${\mathbf{y}}$ is the response variable; $\varvec{m}$ is the overall mean; 1 is a vector of ones; ${\varvec{\upbeta}}$ is the vector of the fixed effects for each breakdown associated with the incidence matrix ${\mathbf{X}}$; ${\mathbf{f}}\left( {\mathbf{t}} \right)$ is a cubic spline for age with smoothing parameter calculated using ASReml and included in the solving algorithm as a random effect; ${\mathbf{u}}$ is the vector of random sire effects with ${\mathbf{u}}\sim{\mathbf{MVN}}\left( {\mathbf0,{\mathbf{A}}\upsigma_{\text{s}}^{2} } \right)$ associated with the incidence matrix ${\mathbf{Z}}$; and ${\mathbf{e}}$ is the residual error with ${\mathbf{e}}\sim{\mathbf{MVN}}\left( {\mathbf0,{\mathbf{I}}\upsigma_{\text{e}}^{2} } \right)$. Heritability for the sire models was calculated as $4\upsigma_{\text{s}}^{2} /\upsigma_{\text{p}}^{2}$, where $\upsigma_{\text{p}}^{2} =\upsigma_{\text{s}}^{2} +\upsigma_{\text{e}}^{2}$. These models were also fitted after removing the reactors under the standard interpretation i.e. *dc* > 4.

#### Correlations and regressions for the components of SICCT

A series of bivariate analyses were conducted following the hierarchy of components of SICCT between *a*_1_ and *b*_1_, *a*_1_ and *a*_2_, *b*_1_ and *b*_2_, *da* and *db* to obtain phenotypic, genetic and environmental correlations. Furthermore, the phenotypic and genetic regressions of *b*_1_ on *a*_1_, *a*_2_ on *a*_1_, *b*_2_ on *b*_1_, and *db* on *da* were calculated since they underlie key assumptions of the comparative test. The models fitted were bivariate extensions of the univariate models (1):

2 $$\left( {\begin{array}{*{20}c} {{\mathbf{y}}_{1} } \\ {{\mathbf{y}}_{2} } \\ \end{array} } \right) = \left( {\begin{array}{*{20}c} {\varvec{m}_{1} 1} \\ {\varvec{m}_{2} 1} \\ \end{array} } \right) + \left( {\begin{array}{*{20}c} {{\mathbf{X}}_{1} } & 0 \\ 0 & {{\mathbf{X}}_{2} } \\ \end{array} } \right)\left( {\begin{array}{*{20}c} {{\varvec{\upbeta}}_{1} } \\ {{\varvec{\upbeta}}_{2} } \\ \end{array} } \right) + \left( {\begin{array}{*{20}c} {{\mathbf{f}}_{1} \left( {\mathbf{t}} \right)} \\ {{\mathbf{f}}_{2} \left( {\mathbf{t}} \right)} \\ \end{array} } \right) + \left( {\begin{array}{*{20}c} {{\mathbf{u}}_{1} } \\ {{\mathbf{u}}_{2} } \\ \end{array} } \right) + \left( {\begin{array}{*{20}c} {{\mathbf{e}}_{1} } \\ {{\mathbf{e}}_{2} } \\ \end{array} } \right) ,$$

where ${\mathbf{u}} = ({\mathbf{u}}_{1}^{\varvec{T}} ,{\mathbf{u}}_{2}^{\varvec{T}} )$ is distributed as ${\mathbf{MVN}}\left( {\mathbf0,{\mathbf{G}} \otimes {\mathbf{A}}} \right)$, where ${\mathbf{G}}$ is the 2 × 2 (co)variance matrix for the two traits in the analysis.

#### Correlation between SICCT positivity and *dc*

A bivariate analysis using model (2) was conducted between the *dc* measurement (mm) in the animals that are assumed healthy, i.e. NR and IR, and the SICCT positivity, which was defined as 1 if R, or 0 if NR or IR under the standard interpretation. The analysis was conducted on the observed scales for both traits. In this analysis, the test measurements for animals classified as reactors were treated as missing.

#### Across-age analyses

Three age groups were defined as follows: Group 1: age ≤ 750 days, Group 2: 750 days < age ≤ 1100 days, and Group 3: age > 1100 days (Table 2). These age groups aim at capturing the pattern observed in the LOESS analysis for animals that were less than 3 years old in the preliminary analysis (see “Results” section), and they approximately correspond to different management groups: for ~25 months average age at first calving (i.e. ~750 days), and a voluntary waiting period of ~50 days, plus ~30 days until second successful conception (age at second calving ~1100 days). Analyses to estimate heritabilities were conducted in ASReml following model (1), for *dc*, *da* and *db*, within each of the three age-groups. In addition, a multivariate analysis was conducted among the three age-groups using a tri-variate extension to model (2), to obtain genetic correlations across different ages.

**Table 2:** Number of records according to age-groups used in the preliminary analysis

	Group 1	Group 2	Group 3	Total
	Age ≤ 750	750 < Age ≤ 1100	Age > 1100	
First records	39,265	16,412	61,665	117,342
(a) R				
* n*	407	443	1854	2704
* dc* (SD)	14.70 (11.61)	10.58 (7.05)	9.78 (6.83)	
(b) NR and IR				
* n*	38,858	15,969	59,811	114,638
* dc* (SD)	−0.15 (1.05)	−0.18 (1.32)	−0.13 (1.32)	

Number of records in each of the age-groups after retaining only the first records where (a) is the number of reactors with *dc* > 4 and with corresponding mean *dc* values for each of the age-groups, and (b) is the number of non-reactors with *dc* ≤ 4 and with corresponding mean *dc* values in each of the age-groups

## Results

### Multiple-test days within breakdowns

**Fig. 3:**
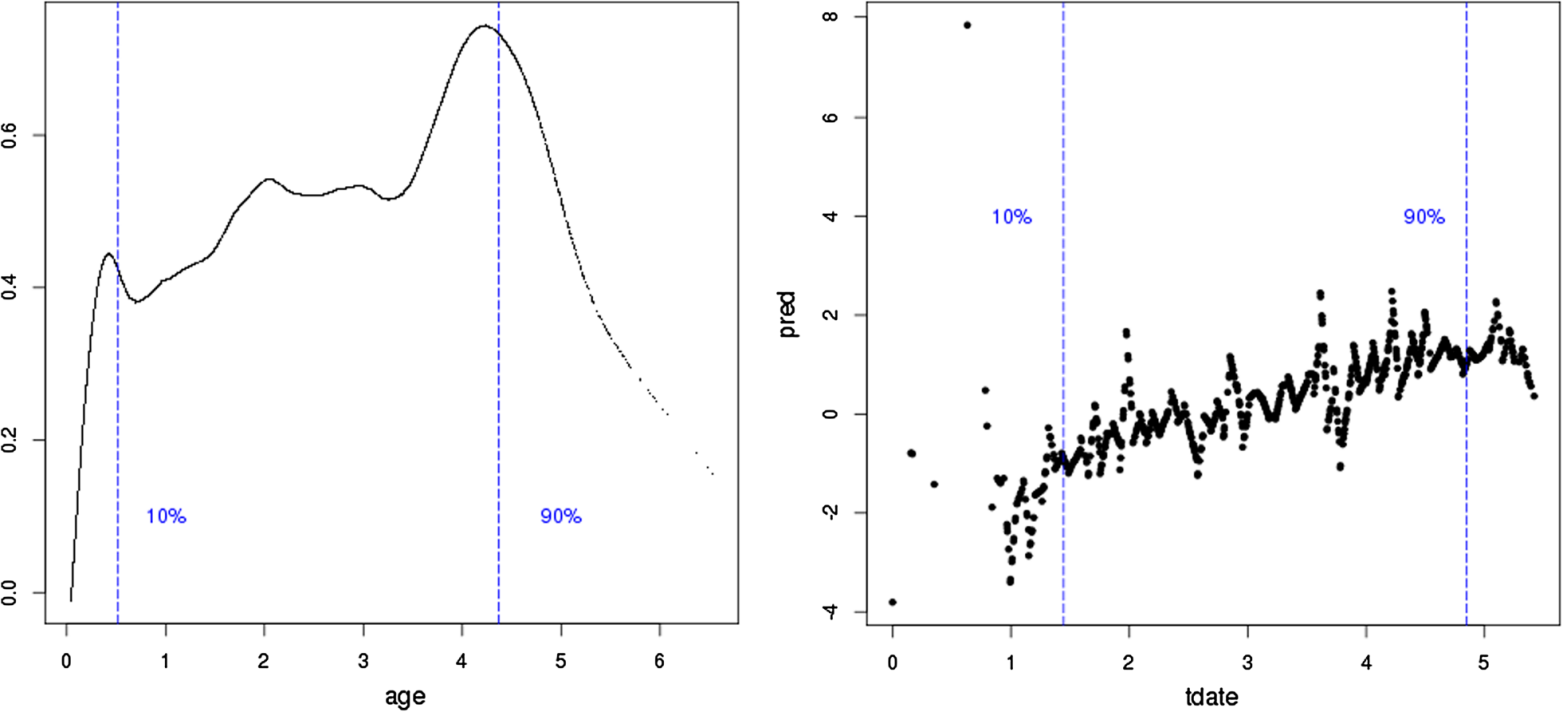
SICCT and test date predicted values. Fitted cubic smoothing spline for the predicted value for *dc* from the ASReml analysis for 100 knot points. *Left* against age, *right* against test date (*tdate*). The 10 and 90% of the data distribution are also shown

The results from fitting models that account for multiple-test days within a breakdown are in Fig. 3. These results indicate that these models were unreasonable since unrealistic time trends for the testing procedure were obtained, i.e. over the course of data collection the predicted values for *dc* increased by 4 units of magnitude. This result indicated a cryptic structure that arises from repeated test dates within breakdowns and justified the restriction to first tests within breakdowns only.

### Variance component analyses

Estimates of heritability after retaining only the first known test within each breakdown and first breakdowns per herd are in Table 3. Both *a*_1_ and *b*_1_ resulted in similar estimated heritabilities of ~0.49 (SE = 0.021), which demonstrates that skin thickness is moderately heritable. Estimated heritabilities for *da* and *db* were very low and similar. Nevertheless, these estimated heritabilities for *da* and *db* were greater than that for *dc* which was estimated as 0.010 (SE = 0.003) and statistically significant (*P* < 0.001). The genetic variance observed for *db* (0.257) was greater than that for *da* (0.038), although the increase in phenotypic variance for *db* resulted in a heritability very close to that for *da*. The large genetic variance for *db* is anticipated to include the variance that comes from the expression of genetic variation in resistance to bTB, amongst other sources. The magnitude of the genetic variances in *da*, *db* and *dc* are biologically relevant although the heritabilities may be small: the genetic standard deviations of *da*, *db*, *dc* were equal to 0.19, 0.50 and 0.14 mm, respectively, which implies that the upper and lower 5% in the population will differ by 0.63 mm in *da*, 0.85 mm in *db* and 0.47 mm in *dc* due to genetic factors. However, it is anticipated that the values for *db* and *dc* in particular would vary with prevalence.

**Table 3:** Estimates of heritability for the components of SICCT

	$\varvec{\sigma}_{\varvec{A}}^{2}$ (SE)	$\varvec{\sigma}_{\varvec{P}}^{2}$ (SE)	$\varvec{h}^{2}$ (SE)
*a*_1_	0.540 (0.026)	1.105 (0.008)	0.489 (0.021)
*b*_1_	0.557 (0.027)	1.128 (0.008)	0.493 (0.021)
*da*	0.155 (0.020)	2.759 (0.013)	0.056 (0.007)
*db*	0.257 (0.035)	4.349 (0.021)	0.059 (0.008)
*dc*	0.038 (0.012)	3.645 (0.017)	0.010 (0.003)

Estimates of heritability after retaining only the first test within each breakdown from fitting Model 1

Estimates of heritability obtained from the univariate analyses after removing the R cases under the standard interpretation, are in Table 4. Removing the R cases had no impact on the estimated heritabilites for *a*_1_, *b*_1_, or *da*. The estimated heritability for *dc* was slightly increased, while that for *db* was slightly reduced compared to the analyses including the R cases, but these differences were within the range of the standard errors of the estimates. The truncation on *dc* caused by removing the R cases had a notable impact on reducing the genetic variance in *db*.

**Table 4:** Estimates of heritability after removing standard R cases

	$\varvec{\sigma}_{\varvec{A}}^{2}$ (SE)	$\varvec{\sigma}_{\varvec{P}}^{2}$ (SE)	$\varvec{h}^{2}$ (SE)
*a*_1_	0.544 (0.026)	1.103 (0.008)	0.493 (0.021)
*b*_1_	0.562 (0.027)	1.128 (0.008)	0.498 (0.022)
*da*	0.132 (0.018)	2.628 (0.013)	0.050 (0.007)
*db*	0.060 (0.009)	1.407 (0.007)	0.043 (0.006)
*dc*	0.029 (0.006)	1.414 (0.007)	0.020 (0.004)

Estimates of heritability after retaining only the first test within each breakdown from fitting Model 1, and after removing the R cases under the standard interpretation

### Correlations between the components of SICCT and regression analyses

The results from the bivariate analyses between *a*_1_ and *b*_1_, *a*_1_ and *a*_2_, *b*_1_ and *b*_2_, and *da* and *db*, are in Table 5. The variances obtained in the bivariate analyses and univariate analyses were similar. In the bivariate analyses, *a*_1_ and *b*_1_ were highly genetically correlated (>0.99) with both genetic and phenotypic regressions of *b*_1_ on *a*_1_ close to 1. This was consistent with expectations since *a*_1_ and *b*_1_ are measurements of the skin thickness on the same animal, thus they measure biologically the same trait on the same scale. *a*_2_ was found to be highly genetically correlated with the initial skin thickness measurement *a*_1_ (0.910, SE = 0.012), and similarly *b*_2_ was highly genetically correlated with *b*_1_ (0.871, SE = 0.018). The genetic and phenotypic regressions for both *a*_2_ on *a*_1_ and *b*_2_ on *b*_1_ were close to but marginally greater than 1, and their standard errors indicated that this difference was statistically significant.

**Table 5:** Estimated parameters from bivariate analyses for the SICCT hierarchy

***A***	***B***	$\varvec{r}_{\varvec{G}}$	$\varvec{r}_{\varvec{P}}$	$\varvec{h}_{{(\varvec{A})}}^{2}$	$\varvec{h}_{{(\varvec{B})}}^{2}$	$\varvec{b}_{\varvec{G}}$	$\varvec{b}_{\varvec{P}}$	$\varvec{\sigma}_{{\varvec{P}(\varvec{A})}}^{2}$	$\varvec{\sigma}_{{\varvec{P}(\varvec{B})}}^{2}$
*a*_1_	*b*_1_	0.999 (0.000)	0.958 (0.000)	0.493 (0.021)	0.493 (0.021)	1.009 (0.005)	0.967 (0.001)	1.106 (0.008)	1.128 (0.008)
*a*_1_	*a*_2_	0.910 (0.012)	0.559 (0.003)	0.489 (0.021)	0.215 (0.013)	1.149 (0.033)	1.064 (0.006)	1.105 (0.008)	4.005 (0.022)
*b*_1_	*b*_2_	0.871 (0.018)	0.477 (0.003)	0.492 (0.021)	0.178 (0.012)	1.168 (0.042)	1.065 (0.008)	1.128 (0.008)	5.623 (0.030)
*da*	*db*	0.901 (0.029)	0.500 (0.003)	0.063 (0.008)	0.053 (0.007)	1.040 (0.071)	0.627 (0.004)	2.763 (0.014)	4.345 (0.021)
*da*	*dc*	0.077 (0.141)	−0.325 (0.003)	0.063 (0.008)	0.012 (0.003)	0.039 (0.070)	−0.373 (0.004)	2.763 (0.014)	3.645 (0.017)
*db*	*dc*	0.500 (0.107)	0.657 (0.002)	0.053 (0.007)	0.012 (0.003)	0.217 (0.056)	0.602 (0.002)	4.345 (0.021)	3.645 (0.017)

Results from the first-records bivariate analysis after retaining only the first known test within each breakdown: $\varvec{r}_{\varvec{G}}$ and $\varvec{r}_{\varvec{P}}$ are the genetic and phenotypic correlations respectively, and $\varvec{b}_{\varvec{G}}$ and $\varvec{b}_{\varvec{P}}$ are the genetic and phenotypic regressions of trait B on trait A, $\varvec{h}_{{(\varvec{A})}}^{2}$ and $\varvec{h}_{{(\varvec{B})}}^{2}$ are the estimated heritabilities and $\varvec{\sigma}_{{\varvec{P}(\varvec{A})}}^{2}$ and $\varvec{\sigma}_{{\varvec{P}(\varvec{B})}}^{2}$ are the phenotypic variances for traits A and B, respectively

The genetic correlation between the derived traits *da* and *db* was positive and large (0.901, SE = 0.029) but it was statistically different from 1, with a genetic regression of *db* on *da* close to 1 (1.040, SE = 0.071). The phenotypic regression of *db* on *da* was notably less than 1 (0.627, SE = 0.004), and much closer to the environmental regression of *db* on *da* i.e. 0.620 (SE = 0.004), since the genetic variance forms only a small part of the total variance in *db* and *da*. The genetic correlations of *dc* with *da* and *db* were equal to 0.077 (SE = 0.141) and 0.500 (SE = 0.107) respectively, with a value close to 0 for the correlation between *da* and *dc*, which is a consequence of the genetic regression of *db* on *da* being close to 1 and of *dc* = *db* − *da.* The small magnitude of the genetic correlation of *dc* with *da* is consistent with the observation that removing the standard R cases, hence truncating the values on *dc*, had only a small impact on the genetic variance in *da* (see Tables 4, 5). The corresponding phenotypic correlations for *dc* with *da* and *db* were equal to −0.325 (SE = 0.003) and 0.657 (SE = 0.002), respectively. The implications of these results are discussed in detail in the “Discussion” section.

The estimated parameters obtained from the bivariate analyses after removing the R cases under the standard interpretation are in Table 6 and reflect the truncation of the distributions on *dc.* Removing the R cases had only a small impact on the genetic correlations but did increase their values towards 1. Post-truncation, the genetic regression of *db* on *da* was less than 1 (0.607 SE = 0.037). As indicated above, truncation on *dc* had a large impact on the genetic variance in *db* and only a small impact on the genetic variation in *da* and post-truncation genetic correlations of *dc* with *da* and *db* were equal to −0.840 (SE = 0.045) and −0.566 (SE = 0.107), respectively.

**Table 6:** Estimated parameters from bivariate analyses for the SICCT hierarchy after removing the standard R

***A***	***B***	$\varvec{r}_{\varvec{G}}$	$\varvec{r}_{\varvec{P}}$	$\varvec{h}_{{(\varvec{A})}}^{2}$	$\varvec{h}_{{(\varvec{B})}}^{2}$_**)**_	$\varvec{b}_{\varvec{G}}$	$\varvec{b}_{\varvec{P}}$	$\varvec{\sigma}_{{\varvec{P}(\varvec{A})}}^{2}$	$\varvec{\sigma}_{{\varvec{P}(\varvec{B})}}^{2}$
*a*_1_	*b*_1_	0.999 (0.001)	0.958 (0.000)	0.498 (0.021)	0.498 (0.021)	1.009 (0.005)	0.968 (0.001)	1.105 (0.008)	1.128 (0.008)
*a*_1_	*a*_2_	0.923 (0.011)	0.567 (0.003)	0.493 (0.021)	0.220 (0.014)	1.153 (0.032)	1.060 (0.006)	1.103 (0.008)	3.865 (0.021)
*b*_1_	*b*_2_	0.960 (0.018)	0.681 (0.003)	0.498 (0.021)	0.284 (0.012)	1.104 (0.042)	1.037 (0.008)	1.128 (0.008)	2.619 (0.015)
*da*	*db*	0.923 (0.023)	0.682 (0.002)	0.052 (0.007)	0.042 (0.006)	0.607 (0.037)	0.499 (0.002)	2.629 (0.013)	1.407 (0.007)
*da*	*dc*	−0.840 (0.045)	−0.684 (0.002)	0.052 (0.007)	0.021 (0.004)	−0.393 (0.037)	−0.502 (0.002)	2.629 (0.013)	1.415 (0.007)
*db*	*dc*	−0.566 (0.107)	0.068 (0.003)	0.042 (0.006)	0.021 (0.004)	−0.403 (0.089)	0.068 (0.003)	1.407 (0.007)	1.415 (0.007)

Results from the first-records bivariate analysis after retaining only the first known test within each breakdown and after removing the R cases under the standard interpretation

### Genetic basis of variation in SICCT response conditional on infection status

The bivariate analysis between the *dc* measurement (mm) in the animals classified as healthy and SICCT positivity (i.e. passing the threshold of being a standard R or not) provided a genetic correlation of −0.01 (SE = 0.14). This value provides evidence of the magnitude of the genetic covariance between SICCT responses in healthy animals and resistance to bTB, but is interpreted in more detail in Additional file 3 and the "Discussion" section. In addition, the standard reactors to the avian tuberculin (i.e. *dc* < −4) were also removed from the data. The genetic correlation between the *dc* measurement and SICCT positivity remained very small (0.07, SE = 0.16).

### Across-age analyses

**Fig. 4:**
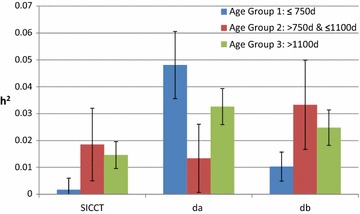
Heritability estimates among different age-groups. Estimated heritabilities for *dc*, *da* and *db*, from the analysis on the first known test within each breakdown, for each of the three age-groups

The results of the analysis within each of the three age-groups, when using the first known test within each breakdown, are in Tables 7, 8, and 9. The genetic variance in *a*_1_ and *b*_1_ is particularly evident at young ages while heritability decreases to very low values in age-groups 2 and 3. The genetic variances for *da*, *db* and *dc* are more consistent across age-groups, with all estimated heritabilities for *dc* being less than 0.02 (Fig. 4). For the second age-group, estimated heritabilities were higher for *dc* and *db* than for *da*.

**Table 7:** Estimated heritabilities for SICCT and its components in age-group 1

	$\varvec{\sigma}_{\varvec{A}}^{2}$ (SE)	$\varvec{\sigma}_{\varvec{P}}^{2}$ (SE)	$\varvec{h}^{2}$ (SE)
*a*_1_	0.742 (0.058)	1.156 (0.016)	0.642 (0.043)
*b*_1_	0.817 (0.061)	1.194 (0.017)	0.684 (0.044)
*da*	0.095 (0.025)	1.964 (0.018)	0.048 (0.013)
*db*	0.030 (0.016)	2.893 (0.026)	0.010 (0.005)
*dc*	0.005 (0.013)	2.983 (0.027)	0.002 (0.004)

Analysis of the estimated heritabilities on the first known test within each breakdown for age-group 1

**Table 8:** Estimated heritabilities for SICCT and its components in age-group 2

	${\varvec{\upsigma}}_{{\mathbf{A}}}^{2}$ (SE)	${\varvec{\upsigma}}_{{\mathbf{P}}}^{2}$ (SE)	${\mathbf{h}}^{2}$ (SE)
*a*_1_	0.089 (0.022)	0.938 (0.013)	0.095 (0.023)
*b*_1_	0.071 (0.020)	0.969 (0.013)	0.073 (0.020)
*da*	0.039 (0.037)	2.882 (0.038)	0.013 (0.013)
*db*	0.157 (0.079)	4.728 (0.062)	0.033 (0.017)
*dc*	0.077 (0.056)	4.145 (0.054)	0.019 (0.014)

Analysis of the estimated heritabilities on the first known test within each breakdown for age-group 2

**Table 9:** Estimated heritabilities for SICCT and its components in age-group 3

	${\varvec{\upsigma}}_{{\mathbf{A}}}^{2}$ (SE)	${\varvec{\upsigma}}_{{\mathbf{P}}}^{2}$ (SE)	${\mathbf{h}}^{2}$ (SE)
*a*_1_	0.074 (0.010)	0.853 (0.006)	0.087 (0.011)
*b*_1_	0.082 (0.010)	0.867 (0.006)	0.094 (0.012)
*da*	0.096 (0.020)	2.941 (0.019)	0.033 (0.007)
*db*	0.115 (0.031)	4.652 (0.029)	0.025 (0.007)
*dc*	0.053 (0.018)	3.660 (0.023)	0.015 (0.005)

Analysis of the estimated heritabilities on the first known test within each breakdown for age-group 3

The genetic correlations obtained from the multivariate analysis across the three age-groups are in Table 10. The correlation between age-groups 1 and 2 was estimated to be on the boundary. The low magnitude of the estimates of genetic variances combined with their lack of precision contributed to the low power for estimating these correlations. This was evident from the likelihood-ratio test statistic (*Δ*), by testing this model against a null hypothesis that all correlations were 0, and a further null hypothesis that all genetic correlations were 0.9: in neither case did the test criterion exceed the 5% significance threshold for $\varvec{\chi}_{3}^{2}$ (7.89): for *r*_*A*_ = 0, *Δ* = 3.98, and for *r*_*A*_ = 0.9, *Δ* = 6.36.

**Table 10:** Analysis across age-groups

	Group 1	Group 2	Group 3
Group 1	1	0.999^a^	−0.643
Group 2	0.999^a^	1	−0.329
Group 3	−0.643	−0.329	1

Genetic correlations for SICCT across the different age-groups from the multivariate analysis

^a^The estimate was on the boundary

## Discussion

Any intervention in bTB control must be examined for its potential impact on existing control measures and SICCT is one of the most important of these measures. Culling of individuals, and restrictions on herd management that arise from bTB controls are strongly associated with the outcome of the SICCT, with ~93% of all bTB-associated culls and bTB-confirmed cases associated with an animal’s response to the SICCT (Banos G., personal communication, November 18th, 2015). Therefore the finding and documentation of genetic variation in the biology of SICCT and its components are important, and the potential consequences need to be considered, with or without artificial selection for resistance to infection. In cattle, there is strong evidence for genetic variation in resistance to bTB infection [4, 5] and any interpretation of this evidence as a differential response to infection in SICCT response, i.e. genetic variation in the individual *Se* to SICCT, is incompatible with the published evidence (see Additional file 4).

There are (at least) three traits for which genetic variation may influence bTB control: (1) the liability to become infected with bTB when exposed to the pathogen; (2) the liability of avoiding positivity (i.e. pass or fail) in the SICCT when not infected with bTB (individual *Sp*—this phrasing is determined by the definition of specificity as the probability of SICCT indicating uninfected given the individual is uninfected); and (3) the liability for positivity in the SICCT test when infected with bTB (individual *Se*). Information on the first of these traits comes from observing cases and survivors over the full course of a breakdown and it is not proposed that the continuous variation of SICCT that is studied here adds significantly to this information. In contrast, genetic variation in the continuous variation in SICCT is the direct measure for the underlying liabilities for an individual *Sp* in healthy animals, as *dc* is the continuous trait upon which the threshold for culling is applied. Interpreting the continuous variation in SICCT in infected animals as a liability for individual *Se* is more complex as the true liability for positivity will depend on the time since infection which is unknown among other factors.

The data used for this study is sufficient in size to have the power to detect and quantify genetic variances that represented ~1% of the phenotypic variance. A further strength of the data is that it is field data, and both genetic and non-genetic influences shaping the data are practically relevant (e.g. the Holstein–Friesian breed under study). However this data also has important weaknesses: (1) it is not possible to infer the disease status of individuals unambiguously so that some individuals may be wrongly considered as healthy, and some may be wrongly considered as infected; and (2) the quality of the recorded data has been questioned [14, 15], and the need for improving the quality of the measurements and the consistency across tests has been recognised by the UK government by introducing further quality controls in future bTB test recording. The magnitude of the uncertainty in disease status is quantified in Additional file 2, and Additional file 3 uses a quantitative model to overcome some of these uncertainties in order to make some inferences feasible.

The data in the present analysis do pass some tests on data quality in that *a*_1_ and *b*_1_ are both measurements of skin thickness in a well-defined location taken prior to inoculation and it is anticipated that the scales of variation would be similar, as was found, and that the genetic correlation would be equal to 1 since they measure the same biological trait. In the data, the observed estimate of this genetic correlation could not be distinguished from 1 despite the significant power of the data. This correspondence was also observed in the phenotypic variation although the phenotypic regression was marginally less than 1, which was expected since some environmental variation, e.g. measurement and recording error, in *a*_1_ will be independent of *b*_1_ and vice versa. However, it is expected that *a*_2_ and *b*_2_ are more prone to poor recording practices with recording by the observer based on the perceived NR/IR/R outcome rather than the measurement itself. Therefore, while the results are an important part of the knowledge base, and as such will influence decision making, they require careful interpretation.

All the models presented were sire models combined with pedigree information. The sire model offers further protection against suboptimal data quality. In the sire model, information is based on variation between sire means which are modified averages of half-sibs, and as a result of the Central Limit Theorem, this model is less vulnerable to the underlying distribution of the variables. Further modelling challenges arise from the structure of the herds and breakdowns in the data (e.g. due to geographic location, management, test operatives). Given the information available, the exact sources of such a structure are unknown and so the breakdown was fitted as a fixed effect, which recovered information from within herds and avoided any inference of genetic variance from between breakdowns.

The primary findings of this study show that the SICCT is genetically robust in that its design provides substantial protection against random genetic changes that arise from genetic drift and from correlated responses among its components due to either natural or artificial selection. Whereas robustness was the intention of a comparative test [16], this was previously considered only at a phenotypic level, and did not consider genetic changes in the populations, and it is well-established that genetic and environmental relationships can be qualitatively different e.g. [17]. This robustness is seen in the hierarchy of contributing measurements within *dc*: (*a*_1_, *a*_2_, *b*_1_, *b*_2_), to (*da*, *db*), to *dc* itself.

The two measurements *a*_1_ and *b*_1_ represent skin thickness measurements at the site of observation and they show substantial genetic variation particularly at young ages within the Holstein–Friesian gene pool (and breed variation will add to this in the wider population). The results show that this genetic variation in skin thickness contributes directly to genetic variation in *a*_2_ and *b*_2_ with high genetic correlations, so that any assessment of *b*_2_ or *a*_2_ as a single measure of bTB or avian infection will be flawed. Such an assessment would be open to substantial genetic drift, irrespective of selection, would likely vary by breed and sub-population, and diagnostic ranges would depend on age. The use of *da* and *db* controls both these risks associated with skin thickness. This was demonstrated through the regressions of *a*_2_ on *a*_1_, and *b*_2_ on *b*_1_ which were found to be close to 1, indicating that by using *da* and *db* the test becomes near-independent of skin thicknesses, *a*_1_ and *b*_1_, i.e. *da* and *db* are not a function of *a*_1_ and *b*_1_ as *da* = *a*_2_–1*.a*_1_ and similarly *db* = *b*_2_–1*.b*_1_. These findings were also found across ages, showing that this control for skin-thickness was not age-dependent although the genetic variance for *a*_1_ and *b*_1_ varied with age. Therefore, including *a*_1_ and *b*_1_ and calculating *da* and *db* make SICCT more stable with respect to the genetics underlying the initial skin thickness.

Similarly, it is important that the genetic regression of *db* on *da* is 1 prior to culling decisions, and the estimated value of 1.03 found here (SE = 0.03) is therefore reassuring. If this regression were different from 1 then correlated responses in SICCT could arise due to changes in specific components. The magnitude of the genetic correlation between *da* and *db* is less important and is not expected to be 1, since the additional genetic variance in resistance to infection, and other possible sources of variation in *db* which are independent of *da*, and in *da* which are independent of *db* will reduce it. Nevertheless, a high genetic correlation might be expected even in the presence of genetic variance in resistance to infection since the correlation will be inversely related to prevalence (i.e. what proportion of animals are expressing the additional variance in *db*) and in this sample the prevalence, while uncertain, is unlikely to be greater than 3% given a fraction of 2.3% of R animals even under the severe interpretation. The implication of the genetic regression of 1 is that if *A*_*db*_ and *A*_*d*a_ are breeding values for *db* and *da* respectively, with phenotypes *db* = *A*_*db*_ + *ε*_*db*_ and correspondingly *da* = *A*_*da*_ + *ε*_*da*_ where *ε* represents deviations of environmental origin, then *dc* is independent of the additive genetic variation in *da* since *A*_*db*_ = *A*_*da*_ + *δ* where *δ* represents genetic terms independent of *A*_*da*_. These results were also evident across age groups, which indicates the robustness of SICCT that arises from the hierarchy. In summary, the observation that there is genetic variance in *da*, with an estimated heritability of ~0.05, and its high genetic correlation with variation in *db* indicate the possibility of relatively weak random genetic drift due to non-specific responsiveness to environmental mycobacteria in non-comparative tests based on *db* alone, but this is effectively removed by the comparative nature of SICCT. A corollary of this finding is that SICCT itself is unlikely to drift across generations through genetic changes in *da* as was proposed [9]. Although observing a low heritability may limit drift in the short- to medium-term, it does not exclude more substantial genetic change as a correlated response to selection on other traits, such as selection on resistance discussed below. For example, traditional measures of fertility in dairy cattle have low heritabilities, yet the strong selection for higher milk yield has reduced fertility as a result of an unfavourable genetic correlation, a trend only recently reversed by using EBV for fertility.

These concepts have implications beyond the genetic variation in bovine SICCT components, i.e. first in the wider conduct of SICCT testing, and second in the search for genetic risks to human TB. The concept that *dc* = *db* − *1*.*da* removes the confounding responses to environmental mycobacteria relies on the regression coefficient of 1: while this was true for the genetic variance, it was not the case for at least some of the non-genetic sources of variance in *dc* as the estimated phenotypic regression was much less than 1, namely 0.63. The hypothesis that arises from this estimate is that the use of *dc* is over-correcting for this non-genetic variance and that animals with a large positive *da* originating from a non-genetic source have a lower risk of being declared as R, which was the implication of the negative phenotypic correlation observed between *dc* and *da*. Such biases have no implications for genetic change. The non-genetic sources of variance encompass measurement and recording errors but this study has no information on which to decompose all the sources of non-genetic variance. The second implication concerns the use of human TB tests for detecting significant quantitative trait loci (QTL) [18–20]. These use results from a non-comparative tuberculin skin test (TST) [21] and in the QTL studies, variation to the TST, which is an analogue to *db*, is interpreted as resistance. The use of non-comparative tests is open to confounding with background genetic variation in response to environmental mycobacteria, as shown in this study. The comparative nature of SICCT removes such ambiguities in cattle.

The more challenging question is what impact selection on predictions of disease resistance, which are made by using models analogous to the ultimate fate models of [5], may have on the efficacy of SICCT as a control measure, given that SICCT and its components exhibit underlying continuous heritable variation. Specifically, the concern is with correlated responses in the individual *Sp* and *Se* leading to changes in population-wide *Sp* and *Se*. Despite the inevitable uncertainty in the classification of animals in this study into infected and uninfected, it is possible to draw some conclusions for two reasons which underpin the quantitative model in Additional file 3. First, the magnitude of these uncertainties is relatively small, due to the low prevalence in the data and the high population-wide *Sp* [22] in this data (see Additional file 2). Second, the ultimate fate models used for evaluating resistance rely on which animals are ultimately culled, or culled and confirmed over the course of the completed breakdown. This process will have much greater *Se* than a single SICCT measurement. In the UK, the breakdown in a herd, with its associated management restrictions, continues until two consecutive tests over a minimum period of 60 days are clear across the herd. The purpose of this process is to identify and remove all infected animals, so that the process has a *Se* of 1, or close to it.

These results do provide evidence that suggest that any response due to cross-reactions in healthy animals that reduces population-wide *Sp* is expected to be weak and slow, and negligible over a small number of generations. The current estimates suggest that increasing resistance is likely to slightly reduce specificity (see Additional file 3) although a small beneficial change cannot be excluded. However, the power of the data does exclude a strong correlated response. The data in this study are particularly limited in addressing changes in population-wide *Se*, since this requires observing cases among those that are not detected within the process of SICCT monitoring. Nevertheless, preliminary results have suggested that such sires of bTB cases appear to be a random sample of bulls from the population and do not appear to be a particular subset of bulls within the spectrum of genetic susceptibility to bTB (Banos G., personal communication, November 18th, 2015).

However, some theoretical risks cannot be excluded and would remain even if selection did not take place. The Holstein cattle breed has a small effective population size due to the widespread use of popular sires and if the genetic variation in these bTB and SICCT related traits were large enough to display significant selection response, direct or correlated, they can also exhibit population changes that arise from genetic drift, which is caused by the unrecognised genetic merits of popular sires with respect to bTB. Both the risks associated with selection and with drift can be managed by continued monitoring of key population data, e.g. SICCT measurements as studied here, the outcome of the confirmation process of cases observed with or without SICCT positivity. With such monitoring, implementing genetic selection for bTB resistance is unlikely to compromise the integrity of the test in the short to medium term. Conceivably over longer time periods, without intervention, some response to selection might occur in the individual *Sp*, or in the individual *Se*. In the latter case, a scenario with reduced prevalence but a greater proportion of non-responding infected individuals cannot be excluded. Such risks can be further reduced by developing more accurate genomic predictors for bTB resistance by expanding the training sets of confirmed cases and survivors of breakdowns [7], since this will reduce the role of SICCT in providing data for genetic evaluations of bTB resistance.

## Conclusions

The hierarchy of SICCT was shown to be important at the genetic level, which makes it a genetically robust test for the purposes of genetic selection for reduced susceptibility to bTB. The continuous variation in SICCT is only lowly heritable and the SICCT outcome in the healthy animals is very weakly genetically correlated with the SICCT positivity, which indicate that selection for individuals more resistant to bTB infection is not likely to change the probability of correctly identifying non-infected animals, i.e. the *Sp* of the test.

## Additional files

**Additional file 1.** The effect of age on *dc* for reactors. Figure S1 showing a LOESS fit to the effect of age on the magnitude of *dc* for reactors.

**Additional file 2.** Assessment of the numbers of false positives and negatives within the data. Published values of *Se* and *Sp* for SICCT tests are used to provide estimates of the numbers of animals in the data that may be false positives and negatives in Table S1 [23].

**Additional file 3.** Inferences on the genetic correlation of susceptibility to bTB infection and SICCT response in healthy animals. Development of a quantitative model, which is summarised in Table S2, describing the impact of genetic variation in individual *Sp* on the genetic correlation of susceptibility to bTB infection and SICCT response in healthy animals [23–27].

**Additional file 4.** Published magnitudes of genetic variation in bTB susceptibility cannot be explained by genetic variation in *Se*. A quantitative argument, which is summarised in Figure S2 and Table S3 demonstrating that genetic variation in *Se* alone cannot explain extent of the observed variation among animals declared to be healthy or diseased [5, 28, 29].
