# Supplementary material for: Quantitative genetic analysis of the bTB diagnostic single intradermal comparative cervical test (SICCT)
Source: Genet Sel Evol. 2016 Nov 24;48:90. doi: 10.1186/s12711-016-0264-3 (PMC5123354; doi:10.1186/s12711-016-0264-3)
Supplement: Supplementary file 1 — Additional file 1. The effect of age on dc for reactors. Figure S1 showing a LOESS fit to the effect of age on the magnitude of dc for reactors. [file 12711_2016_264_MOESM1_ESM.pdf]

Additional file 1

The effect of age on dc for reactors.

Figure S1. LOESS analysis of reactors in relation to age.

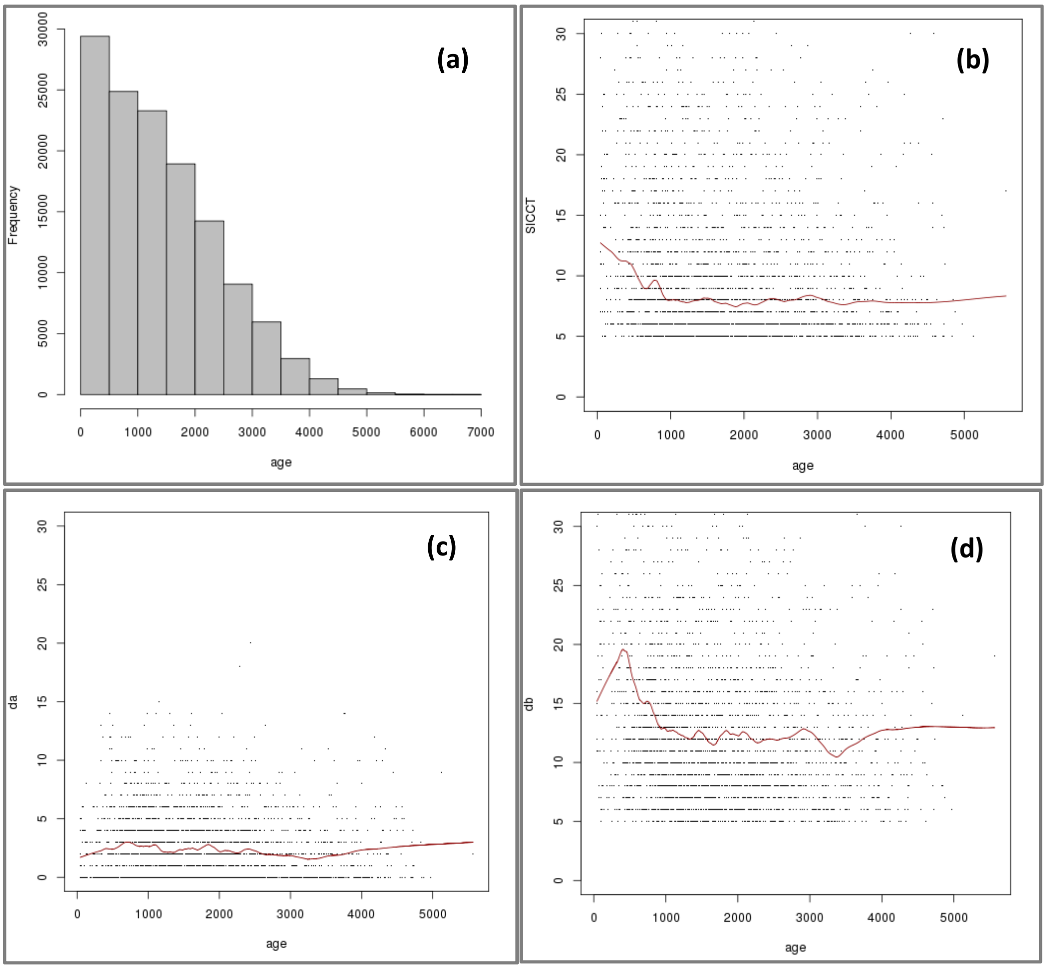

(a) Age distribution in the data; (b) relationship of *dc* with age and fitted line using LOESS for the reactors; (c) relationship of *da* with age and fitted line using LOESS for the reactors; (d) relationship of *db* with age and fitted line using LOESS for the reactors; where reactors are defined under the standard interpretation with SICCT>4.
