# Supplementary material for: Quantitative genetic analysis of the bTB diagnostic single intradermal comparative cervical test (SICCT)
Source: Genet Sel Evol. 2016 Nov 24;48:90. doi: 10.1186/s12711-016-0264-3 (PMC5123354; doi:10.1186/s12711-016-0264-3)
Supplement: Supplementary file 2 — Additional file 2. Assessment of the numbers of false positives and negatives within the data. Published values of Se and Sp for SICCT tests are used to provide estimates of the numbers of animals in the data that may be false positives and negatives in Table S1 [23]. [file 12711_2016_264_MOESM2_ESM.pdf]

Additional file 2

Assessment of the numbers of false positives and negatives within the data.

The data used for detailed analysis contained 89,243 individuals: 1243 presumed cases (R's) and 88,000 presumed healthy (NR+IR), with an apparent prevalence p' = 1.4%. Table S1 considers the 2x2 contingency table classifying diseased and test results for two values of Se for a single SICCT test, 0.55 and 0.65, which are in the lower range of published values [1] and two values of Sp, 0.9998 and 0.9991. The values for Sp are those obtained for the standard and severe interpretations of SICCT taken from [23]. It shows the false positives are likely to be very few in number and unlikely to exceed 6.1% of the observed R (positives), and the false negatives are unlikely to exceed 1.4% of the combined NR and IR (negatives).

Table S1. The distribution of true and false positives for different values of *Se* and *Sp*. The true prevalence is estimated using the standard epidemiological formula $p_{TB} = (p' + Sp - 1)/(Se + Sp - 1)$. The % given corresponds to the total number of 88,932 individuals. (a) *Se* = 0.55, Sp =0.9991 and p_{TB} = 0.0266; (b) *Se* = 0.55, *Sp* = 0.9998 and p_{TB} = 0.0280; (c) *Se* = 0.65, *Sp* = 0.9991 and p_{TB} = 0.0222; (d) *Se* = 0.65, Sp = 0.9998 and p_{TB} = 0.0233. SICCT positivity is '0' if NR or IR and '1' if R.

	SICCT Positivity				
	'0'	%	'1'	%	Total
(a)					
Healthy	86703	97.5	78	0.1	86781
Diseased	968	1.1	1183	1.3	2151
Total	87861		1261		88932
(b)					
Healthy	86653	97.4	17	0.0	86671
Diseased	1018	1.1	1244	1.4	2261
Total	87861		1261		88932
(c)					
Healthy	87034	97.9	78	0.1	87113
Diseased	637	0.7	1183	1.3	1819
Total	87861		1261		88932
(d)					
Healthy	87001	97.8	17	0.0	87018
Diseased	670	0.8	1244	1.4	1914
Total	87861		1261		88932
