# Supplementary material for: Quantitative genetic analysis of the bTB diagnostic single intradermal comparative cervical test (SICCT)
Source: Genet Sel Evol. 2016 Nov 24;48:90. doi: 10.1186/s12711-016-0264-3 (PMC5123354; doi:10.1186/s12711-016-0264-3)
Supplement: Supplementary file 3 — Additional file 3. Inferences on the genetic correlation of susceptibility to bTB infection and SICCT response in healthy animals. Development of a quantitative model, which is summarised in Table S2, describing the impact of genetic variation in individual Sp on the genetic correlation of susceptibility to bTB infection and SICCT response in healthy animals [23–27]. [file 12711_2016_264_MOESM3_ESM.pdf]

Additional file 3

Inferences on the genetic correlation of susceptibility to bTB infection and SICCT response in healthy animals.

As reported in the Materials & Methods and Results, a bivariate analysis was carried out on SICCT positivity and the *dc* value for all animals with score 0 for SICCT positivity, i.e. animals considered healthy. The appearance of false negatives among those considered healthy are neglected in this analysis since they contribute only 1.1% of animals with score 0 assuming a *Se* for a SICCT measurement of 0.55 for standard interpretation, and *Sp* = 0.9998 [23] (see Supplementary Information 2). Likewise the deletion of the small number of healthy animals with standard positivity (see Supplementary Information 2) can be neglected since they represent a negligible selection intensity within uninfected animals: assuming the *Sp* of [23] of 0.9998 for the standard SICCT interpretation, the standardised selection intensity is 0.0008. Villanueva and Kennedy [24] show that the impact of such weak selection on the genetic correlation is negligible. For healthy animals, *dc* is the underlying liability phenotype of SICCT positivity. Therefore the observed genetic correlation may be interpreted as an estimate of the genetic correlation between individual *Sp* on the liability scale and susceptibility to bTB infection on the 0/1 scale, but subject to a bias. This bias is quantified below.

Consider a model with genetic variation in resistance to infection and in individual *Sp*: therefore an individual i has a probability p_i of becoming infected and probability Sp_i of displaying SICCT positivity when not infected. Table S2 shows the standard epidemiological contingency table for SICCT outcomes for this model.

Table S2. The standard epidemiological contingency table modified to account for individual differences in resistance to infection and specificity.

	SICCT positivity '0'	'1'
Healthy	$(1-p_i)\,Sp_i$	$(1-p_i)\,(1-Sp_i)$
Diseased	$p_i\,(1-Se)$	$p_i\,Se$

For individual *i*, let $p_i = p + \delta_{1,i}$ and $Sp_i = Sp - \delta_{2,i}$ on the 0/1 scale, where $\delta_{1,i}$ and $\delta_{2,i}$ are deviations in breeding value for *i* and where $\delta_{2,i}$ is –ve so that it increases as Sp_i decreases, in concordance with the bivariate analysis presented. Therefore, the probability of SICCT positivity for *i* is:

$$p_i S_e + (1-p_i)(1-Sp_i)$$

$$= [pSe + (1-p)(1-Sp)] + Se\delta_{1,i} + (1-p)\delta_{2,i} - (1-Sp)\delta_{1,i} - \delta_{1,i}\delta_{2,i}$$

$$\approx [pSe + (1-p)(1-Sp)] + (Se + Sp - 1)\delta_{1,i} + (1-p)\delta_{2,i}$$

after neglecting second order terms. Then the probability deviation due to genetics for SICCT positivity for *i* is:

$$\delta_{0,i} = (Se + Sp - 1)\delta_{1,i} + (1-p)\delta_{2,i} \qquad [1]$$

These deviations on the 0/1 scale can be related to the deviations in breeding value on the liability scale by using the result of Robertson [25] in regressing a 0/1 selection score *S* on an underlying phenotype (*L*) after truncation selection with upper tail probability *q* assuming a Normal distribution. Robertson showed the regression coefficient was ϕ_q denoting the normal density function at the truncation point for probability *q*. Further, since the breeding value on the liability scale influences selection score through the phenotype, this is also the regression coefficient of the selection score on the breeding value. These results are used extensively to predict rates of

inbreeding accurately [26, 27]. Therefore deviation $\delta_{1,i} = \phi_p A_{1,i}$ and $\delta_{2,i} = \phi_{Sp} A_{2,i}$ where p denotes prevalence, Sp denotes the population-wide specificity, and $A_{1,i}$, $A_{2,i}$ are breeding values on the liability scale for becoming infected and SICCT positivity when healthy respectively.

The genetic correlation in the bivariate analysis is a correlation of $A_{0,i}$ the breeding value on the 0/1 scale underlying the observation of SICCT positivity, and $A_{2,i}$ the breeding value of the liability for SICCT positivity in healthy animals. The terms relating $A_{0,i}$ to liability in susceptibility to infection and individual Sp can now be calculated by substitution into (1) to give $A_{0,i} = (Se + Sp - 1)\phi_p A_{1,i} + (1-p)\phi_{Sp} A_{2,i}$ Without loss of generality, assume the liabilities have variance 1, so that $var(A_{1,i}) = h_1^2$, $var(A_{2,i}) = h_2^2$ and $cov(A_{1,i}, A_{2,i}) = h_1 r_A h_2$ where r_A is the true genetic correlation between the liabilities for Sp_i and p_i, then:

$$\mathrm{var}(A_{0,i}) = (Se + Sp - 1)^2 \phi_p^2 h_1^2 + 2(Se + Sp - 1)(1-p)\phi_p \phi_{Sp} h_1 r_A h_2 + (1-p)^2 \phi_{Sp}^2 h_2^2$$

$$cov(A_{0,i}, A_{2,i}) = (Se + Sp - 1)\phi_p h_1 r_A h_2 + (1-p)\phi_{Sp} h_2^2 \,.$$

A test of this model can be made for a fixed Sp and Se as p tends to 0: it is predicted the observed correlation will tend to 1 irrespective of r_A, i.e. there will be strong bias. This is reasonable as the data becomes uninformative for the true r_A, as there are no cases, and what is observed is the genetic correlation for individual Sp expressed on the 0/1 scale and on the continuous scale, which will be near perfect for low heritabilities on which the approximation is based.

The bias for the bTB scenario can be predicted by using established values: h^2_1 = 0.18 from [5]; h^2_2 is the heritability of *dc,* taken as 0.01 from this study; the Sp for standard interpretations of 0.9998 was taken from [23]. The Se for a single SICCT test with standard interpretation was varied as 0.4, 0.5 or 0.6 but the outcomes were not qualitatively different. Substituting these values with Se = 0.55 it was found the bivariate analysis with the standard interpretation has only a very small bias across all values of r_A, and the value of -0.01 observed would have been obtained with a true value of r_A =

-0.02. This is small in magnitude albeit slightly deleterious i.e. indicating a small and very weak potential correlated response of reducing breeding values for individual *Sp* from reducing the genetic liability to become infected with bTB. The observed genetic correlation had approximate 95% support intervals of (-0.30, 0.27), i.e. ranging from moderate deleterious to moderately beneficial.
