# Supplementary material for: Quantitative genetic analysis of the bTB diagnostic single intradermal comparative cervical test (SICCT)
Source: Genet Sel Evol. 2016 Nov 24;48:90. doi: 10.1186/s12711-016-0264-3 (PMC5123354; doi:10.1186/s12711-016-0264-3)

Additional file 4

Published magnitudes of genetic variation in bTB susceptibility cannot be explained by genetic variation in Se.

Published evidence of genetic variance in susceptibility to bTB infection comes from data on SICCT +ve animals in closed breakdowns which were subsequently confirmed as infected with *M. bovis* [5]. In such a study there are 3 groups of animals as shown in Fig. S2: A, those identified as truly infected animals; B those that were truly infected but were not identified because of imperfect *Se* in SICCT; and C, uninfected animals. The study found genetic variance in the liability to infection by contrasts of animals in group A with animals in groups B and C, as B and C could not be distinguished in the field data.

Hypothetically, it might be proposed that what is observed is wholly due to genetic variation in the response of animals to infection, i.e. genetic variation in the individual *Se*, and arises from the contrasts involving group A with group B. If this were the case then group C, the uninfected, acts as noise in the detection of genetic variance. Such a scenario is directly analogous to the model of Bishop and Woolliams [28] involving exposure. In this analogy, the exposure (*e*) defined in [28] identifies those animals that are informative (i.e. had actually been exposed to a pathogen) corresponds to the true prevalence of bTB in this scenario (p_{TB}), and the true prevalence among those exposed (*p*) in [28] corresponds to *Se* in the bTB scenario.

Bishop and Woolliams [28] show that *k*, the fractional bias in the heritability of liability due to incomplete exposure, is given by $k = \grave{o}^2\phi_p^2 / \phi_{\grave{o}p}^2$ where $k = 1$ represents no bias, and ϕ_x is the normal density at a truncation point with upper-tail probability *x*. For the bTB scenario this is $k = p_{TB}^2\phi_{Se}^2 / \phi_{p_{TB}Se}^2$. Table S3 gives some sample outcomes for genetic variation observed in the analyses of [5] if it arose solely from genetic variation in individual *Se* and not in bTB resistance for different values of p_{TB} and *Se*. Given the heritability of bTB resistance of 0.18 (s.e. 0.04) [5], the hypothetical argument of genetic variance solely arising from individual *Se* cannot be sustained and

the observed variance must be derived from contrasts involving groups A and C. *Hence the genetic variance involves contrasts of confirmed infected and uninfected and must encompass genetic variance in resistance to infection.*

Table S3. Heritability expected on the liability scale (h^2_L) if genetic variance arose solely from infected animals for two values of apparent prevalence (p'), where $p' = 0.023$ is the apparent prevalence in this dataset under standard interpretation. The *Se* refers to the sensitivity of the breakdown process in identifying bTB infected animals. p_{TB} was calculated from p' assuming Sp = 0.9998 for the standard interpretation [23] and $p_{TB} = (p' + Sp - 1)/(Se + Sp - 1)$.

Se	$p' = 0.014$<br>$h^2_L = 0.2$	$h^2_L = 0.5$	$p' = 0.040$<br>$h^2_L = 0.2$	$h^2_L = 0.5$
0.65	0.010	0.025	0.014	0.035
0.75	0.006	0.014	0.008	0.019

The calculation shown in Table S3 requires parameters for p_{TB} and population mean *Se.* The relevant *Se* is the population-wide *Se* for the control process involving repeated testing, and the process is designed to have this value close to 1. A very conservative lower bound can be obtained from the 33% recurrence rate of breakdowns in Wales over 2 years, indicating a minimum *Se* of the control process of 0.67 [29]. It is conservative as it encompass new infections over the 2 years beyond the end of the breakdown from all sources, including the environmental reservoir. Thus, 0.65 given in Table 1 is likely to be a severe underestimate of *Se* of the control process immediately following the breakdown. The formulae used to calculate true prevalence, p_{TB}, from apparent prevalence is a standard epidemiological formula $p_{TB} = (p' + Sp - 1)/(Se + Sp - 1)$, and Sp = 0.9991, is the value for standard SICCT interpretation given by [23]. Increased severity of interpretation of SICCT decreases *Sp*, and values in Table S3 show small decreases.

Figure S2. A schematic for different groups of animals within a bTB breakdown. The arrows denote contrasts that exist to infer genetic variance in liability, either in resistance to infection (A to C) or to individual *Se* (A to B).

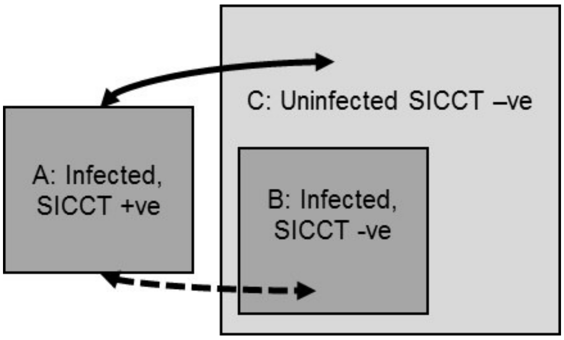

Supplement: Supplementary file 4 — Additional file 4. Published magnitudes of genetic variation in bTB susceptibility cannot be explained by genetic variation in Se. A quantitative argument, which is summarised in Figure S2 and Table S3 demonstrating that genetic variation in Se alone cannot explain extent of the observed variation among animals declared to be healthy or diseased [5, 28, 29]. [file 12711_2016_264_MOESM4_ESM.pdf]
